# Measurement invariance across gender and age groups, validity and reliability of the Chinese version of the short-form supportive care needs survey questionnaire (SCNS-SF34)

**DOI:** 10.1186/s12955-020-01289-0

**Published:** 2020-02-17

**Authors:** Edmond Pui Hang Choi, Qiuyan Liao, Inda Soong, Karen Kar Loen Chan, Conrad C. Y. Lee, Alice Ng, Wing Kin Sze, Janice Wing Hang Tsang, Victor Ho Fun Lee, Wendy Wing Tak Lam

**Affiliations:** 1 School of Nursing, LKS Faculty of Medicine, The University of Hong Kong, Hong Kong, China; 2 School of Public Health, LKS Faculty of Medicine, The University of Hong Kong, Hong Kong, China; 3 Department of Clinical Oncology, Pamela Youde Nethersole Eastern Hospital, Chai Wan, Hong Kong, China; 4 Department of Obstetrics & Gynaecology, Queen Mary Hospital, Hong Kong, China; 5 Department of Clinical Oncology, Princess Margaret Hospital, Hong Kong, China; 6 Department of Clinical Oncology, Tuen Mun Hospital, Hong Kong, China; 7 Department of Clinical Oncology, Queen Mary Hospital, Hong Kong, China; 8 Jockey Club Institute of Cancer Care, Centre for Psycho-Oncology Research & Training, Hong Kong, China; 9 School of Public Health, The University of Hong Kong, Hong Kong, China

**Keywords:** Measurement invariance, Reliability, Unmet need, Validity

## Abstract

**Background:**

Despite the wide use of the Short-Form Supportive Care Needs Survey Questionnaire (SCNS-SF34), the measurement invariance of the SCNS-SF34 across the main groups—gender and age—which might be of interest in the application of the instrument has never been confirmed. To provide an accurate assessment tool to evaluate the unmet needs of Chinese cancer patients, the present study aimed to assess the measurement invariance of the SCNS-SF34 across gender and age groups and to assess the validity and reliability of the Chinese version of the SCNS-SF34.

**Methods:**

The SCNS-SF34 was administrated to 1106 Chinese cancer patients. Other instruments included the Memorial Symptom Assessment Scale-Short Form (MSAS-SF), the Short-Form-12 Health Survey version 2 (SF-12 v2) and the Hospital Anxiety and Depression Scale (HADS). Factor structure, internal construct validity, convergent validity, known-group validity and internal consistency were assessed.

**Results:**

Our data fit the original five-factor model. Multi-group confirmatory factor analysis indicated measurement invariance across age and gender groups. The domains of the SCNS-SF34 had moderate correlations with the corresponding domains of the MSAS-SF, the SF-12 v2 and the HADS, which supported convergent validity. Of the 34 items, 33 had an item-total correlation that was corrected for an overlap of > 0.4 to support the internal construct validity. The SCNS-SF34 aptly differentiated patients by age and gender. The Cronbach’s alpha coefficient ranged from 0.64 to 0.87.

**Conclusions:**

We confirm the measurement invariance of the Chinese version of the SCNS-SF34 across gender and age group. It is a valid and reliable tool for evaluating the needs of Chinese patients with cancer**.**

## Background

Optimising cancer patient-centred care is essential to understand the supportive care needs of cancer patients and to identify any unmet needs [1]. Higher levels of unmet supportive care needs are significantly correlated with more severe levels of psychological distress and poorer health-related quality of life (HRQOL) [2], which leads to higher health service utilisation and costs [3, 4]. Moreover, information on the unmet needs of cancer patients can instigate improvements in health services [1]. Although the unmet supportive care needs of patients with cancer have been assessed in different populations [1], such information about Chinese cancer populations is still limited even though the Chinese represent 20% of the global figure. Previous studies on Chinese populations were mainly conducted on patients with colorectal cancer and breast cancer [5, 6]. That might hinder our understanding of this topic in a broader sense. Thus, there is a need for more research on supportive care needs among Chinese populations.

Several patient-reported outcomes (PROs) have been developed to evaluate the supportive care needs of cancer patients [7]. The Short-Form Supportive Care Needs Survey Questionnaire (SCNS-SF34), which consists of 34 items, is a commonly used instrument. There are 5 domains, including physical and daily living needs (PDL), psychological needs (PSY), patient care and support needs (PCS), health systems and information needs (HSI) and sexuality needs (SEX). The SCNS-SF34 has been validated in different cancer populations, such as China [8], France [9] and Mexico [10]. In Chinese populations, the construct validity and reliability have been established in women with breast cancer in Hong Kong [8] and patients with colorectal cancer in Hong Kong and Taiwan [11] only.

PROs are used to measure latent variables. They can be used to compare groups on some phenomena, such as the level of unmet needs and cancer burden. To be valid for comparison, a PRO must measure identical constructs with the same factor structure across different groups (e.g., gender and age). Evaluating the measurement invariance of PROs across groups of interest can serve the purpose. A good PRO should be able to demonstrate that respondents, across groups of interest, interpret the question items, as well as the underlying construct, in the same way. On the contrary, if measurement invariance cannot be confirmed, mean scores cannot be compared meaningfully. It is because groups or individuals probably interpret the question items differently.

Despite the widespread use of the SCNS-SF34 across different cancer populations, the measurement invariance of the instrument has not been established. A valid comparison of the supportive care needs across age or gender groups requires that the instrument be comparable in these groups. To strengthen and build on the findings of previous validation studies of SCNS-SF34 among cancer patients, the present study aimed to assess the measurement invariance of the SCNS-SF-34 across gender and age groups and to validate the SCNS-SF34 among Chinese cancer patients in Hong Kong by evaluating the psychometric properties.

## Methods

### The study sample and setting

A convenience sample of Chinese patients with cancer was recruited in five public hospitals in Hong Kong. The inclusion criteria were as follows: age ≥ 18 years, within 6 months of completing primary and adjuvant treatment, and fluency in Chinese language. Patients were excluded if they had hearing impairment, refused to join, or were too ill to give informed consent or complete the questionnaires. Patients who consented to join the study were asked to complete a structured questionnaire. A cancer diagnosis and the stages of cancer were retrieved from each patient’s medical record. Ethics approval (UW10–203) was obtained. Written informed consent was obtained for each participant.

### Study instruments

#### SCNS-SF34

The Chinese version of the SCNS-SF34 was used in the present study [12]. The SCNS-SF34 consists of five question areas: the 5-item PDL, the 10-item PSY, the 5-item PCS, the 11-item HSI and the 3-item SEX. Patients rated the intensity of each specified need over the past month on a 5-point Likert scale (1   =  no need, not applicable; 2  =   no need, satisfied; 3   =   low need; 4  =   moderate need; 5  =  high need). It is recommended to use a Likert summated scale by summing the individual items within a domain (item 1 to 5 for the PDL; item 6 to 14, & item 17 for the PSY; item 15, 16 and 31 for the SEX; item 18 to 22 for the PCS; item 23 to 30 and item 32 to 34 for the HSI). Domain scores were converted to standardised Likert summated scores, ranging from 0 to 100, with higher scores indicating a higher perceived unmet care need [13].

#### Memorial symptom assessment scale-short form (MSAS-SF)

The Chinese version of the MSAS-SF was used to measure symptom distress. The first part of the instrument measures distress regarding 28 physical and psychological symptoms during the past week. Patients rate the level of distress of each symptom on a 5-point Likert scale. The second part of the instrument measures the frequency of four psychological symptoms during the past week. Patients rate the frequency of each symptom on a 4-point Likert scale. Only the Physical Symptoms Subscale (MSAS-PHYS) and the Psychological Symptoms Subscale (MSAS-PSYCH) were used in the present study. The subscale scores range from zero to four, with higher scores indicating a higher level of distress. The instrument has been validated in cancer patients in Hong Kong [14].

#### Short-Form-12 health survey version 2 (SF-12 v2)

The Hong Kong Chinese version of the SF-12 v2 was used in this study to measure generic HRQOL during the previous 4 weeks. It consists of scores from the Physical Component Summary and the Mental Component Summary (MCS), with higher scores indicating better generic HRQOL. The psychometric properties of the Chinese version of the SF-12 v2 have been confirmed [15]. It has been widely used for patients with different types of cancer, such as prostate [16].

#### Hospital anxiety and depression scale (HADS)

The Chinese version of the HADS was used in this study. It consists of two subscales, which measure depression and anxiety symptoms during the previous week [17]. Each subscale has seven questions, and patients need to indicate the severity of each symptom on a four-point Likert scale. The score of each item in each subscale is added to generate a total score, ranging from 0 to 21. Higher scores imply more severe anxiety or depression. A review article suggested that the HADS had adequate psychometric properties to assess the severity of symptoms in different populations [18]. Additionally, the HADS has been validated in Hong Kong [19].

### Statistical analysis

#### Factor structure and measurement invariance

First, confirmatory factor analysis (CFA) was used to evaluate the factor structure of the SCNS-SF34. We fit our data to the original five-factor model of the SCNS-SF34 [20]. The CFA was tested using the weighted least squares mean and variance that accounted for the categorical nature of the items. Criteria for an acceptable fit were a root mean square error of approximation (RMSEA) of < 0.06, a comparative fit index (CFI) and a Tucker-Lewis index (TLI) of ≥0.90 [21, 22].

Second, the following steps were conducted with weighted least squares estimator using the theta parameterisation to evaluate the measurement invariance across gender and age groups [23].

1. Thresholds and factor loadings were free across groups. Residual variances were fixed at one in all groups, and factor means were fixed at zero in all groups. It was the least constrained model.
2. Thresholds and factor loadings were constrained to be equal across groups. As a default, residual variances were fixed at one in the first group and freely estimated in the second group. Factor means were fixed at zero in the first group and freely estimated in the second group. It was the more constrained model.

Model difference testing was conducted using the “DIFFTEST” provided by Mplus, which calculated the chi-square difference between the least and more constrained models based on scaling correction factors. If the chi-square difference value was not statistically significant, it indicated that constraining the parameters of the nested model did not significantly worsen the fit of the model, supporting the measurement invariance of the parameters constrained to be equal in the nested model.

#### Internal construct validity and convergent validity

The item-total correlation that was corrected for overlap was used to evaluate internal construct validity. A correlation coefficient of ≥0.4 was used as the threshold for adequate correlation [24]. Convergent validity was assessed using Pearson’s correlations between the SCNS-SF34, HADS, MSAS-SF and SF-12 v2 scores. It was first hypothesised that the PSY domain of the SCNS-SF34 would have a moderate correlation with the HADS, MSAS-PSYCH and SF-12 v2 MCS scores because they specifically measure psychological constructs. It was also hypothesised that the PDL domain of the SCNS-SF34 would have a moderate correlation with the MSAS-PHYS and SF-12 v2 Physical Component Summary scores because they specifically measure constructs that are related to physical aspects.

#### Known-group validity

Four known-group comparisons were carried out using an independent t-test. We first compared the SCNS-SF34 scores between participants ≤60 and > 60 years old. It was hypothesised that younger patients would report higher levels of unmet needs than would older patients [25]. We then compared the SCNS-SF34 scores by gender. Cohen’s D effect sizes were calculated as either trivial (< 0.2), small (≥ 0.2 and < 0.5), moderate (≥ 0.5 and < 0.8) or large (≥ 0.8) [26].

#### Reliability

The internal consistency of the SCNS-SF34 was assessed by Cronbach’s alpha, and a coefficient of ≥0.7 was considered good [27].

Mplus (version 7.4 for Windows) and SPSS (version 23 for Windows) were used.

## Results

### Participants’ characteristics

In total, 1106 cancer patients were included in the analysis. Six hundred and fifty- five participants (59.22%) were female. The mean age was 55.41 years (standard deviation [SD]: 11.91). Eight hundred and twenty-eight participants (74.86%) were either married or cohabitating. More than half the study sample had received either secondary or tertiary education. 30.74% of the participants had a full-time job. More than half of the study sample had a total family income that was ≤ HKD$20,000. The most common type of cancer diagnosis was breast cancer (34.63%), followed by head and neck cancer (19.71%), colorectal cancer (12.03%) and gynaecological cancer (11.66%). In addition, 224, 366, 332 and 81 participants were diagnosed with stages I, II, III and IV cancers, respectively. These results are shown in Table 1.

**Table 1:** Socio-demographic and clinical characteristics

Mean age (SD), n=1103	55.41 (11.91)	Type of cancer, n (%)		Mean score of study outcomes (SD)	
Gender		Breast cancer	383 (34.63)	***SCNS-SF34***	
Male	451 (40.78)	Colorectal cancer	133 (12.03)	Physical and daily living needs (PDL), n=1100	14.75 (14.49)
Female	655 (59.22)	Gynaecological cancer	129(11.66)	Psychological needs (PSY), n=1084	12.42 (13.96)
Marital status, n (%)		Prostate cancer	75 (6.78)	Sexual need (SEX), n=1102	5.29 (11.46)
Single	120 (10.85)	Lung cancer	72 (6.51)	Patient care and support needs (PCS), n=1098	22.97 (20.46)
Married/cohabited	828 (74.86)	Head and neck cancer	218 (19.71)	Health systems and information needs (HIS), n= 1086	34.56 (21.46)
Divorced/separated	87 (7.87)	Leukaemia/lymphoma	25 (2.26)	***SF-12 v2***	
Widowed	68 (6.15)	Sarcoma	6 (0.54)	Physical Component Summary (PCS), n=1068	46.68 (8.45)
Missing	3 (0.27)	Hepatobiliary cancer	8 (0.72)	Mental Component Summary (MCS), n=1068	44.82 (10.74)
Education level, n (%)		Gastric cancer	21 (1.30)	***MSAS-SF***	
No formal education	60 (5.42)	Oesophageal cancer	15 (1.36)	Physical Symptom subscale, n=1096	0.47 (0.52)
Primary education	318 (28.75)	Others	21 (1.90)	Psychological Symptom subscale, n=1092	0.52 (0.69)
Secondary education	577 (52.17)	Stage of disease, n (%)		***HADS***	
Tertiary education	149 (13.47)	Stage I	224 (20.25)	Anxiety level, n=1100	2.32 (2.97)
Missing	2 (0.18)	Stage II	366 (33.09)	Depression level, n=1098	3.06 (3.27)
Occupation, n (%)		Stage III	332 (30.02)		
Full-time	340 (30.74)	Stage IV	81 (7.32)		
Part-time	48 (4.34)	Missing	103 (9.31)		
Retired	291 (26.31)				
Housewife	128 (11.57)				
Unemployed (before diagnosis of cancer)	28 (2.53)				
Unemployed (since diagnosis of cancer)	268 (24.23)				
Missing	3 (0.27)				
Monthly household income, n (%)					
≤ HKD$20,000	658 (59.49)				
> HKD$20,001	380 (34.36)				
Missing	68 (6.15)				

Abbreviation *SD: Standard deviation; SCNS-SF34: the Short-Form Supportive Care Needs Survey Questionnaire; PDL: Physical and daily living needs; PSY: Psychological needs; SEX: Sexual need; PCS: Patient care and support needs: HIS: Health systems and information needs: SF-12 v2: the Short-Form-12 Health Survey version 2; MSAS-SF: the Memorial Symptom Assessment Scale-Short Form; HADS: the Hospital Anxiety and Depression Scale*

The descriptive statistics of the SCNS-SF34 are shown in Table 2. The mean score was 14.75 (SD: 14.49) for the PDL domain, 12.42 (SD: 13.96) for the PSY domain, 5.29 (SD: 11.46) for the SEX domain, 22.97 (SD: 20.46) for the PCS domain and 34.56 (SD: 21.46) for the HSI domain.

**Table 2:** Descriptive statistics, factor structure, internal construct validity and reliability of the SCNS-SF34

		Corrected item-total correlation	Mean (SD)	Floor score^a^ n (%)	Ceiling score^a^ n (%)	No need n (%)	Low unmet need n (%)	Moderate to high unmet need n (%)	Missing value n (%)	Rank, unmet need ^b^
Physical and daily living needs (PDL)			14.75 (14.49)	168 (15.19%)	1 (0.09%)				6 (0.54%)	
1	Pain	0.44	1.70 (0.87)			1023 (92.50%)	20 (1.81%)	63 (5.70%)		21
2	Lack of energy / tiredness	0.54	1.69 (0.76)			1047 (94.67%)	15 (1.36%)	44 (3.98%)		26
3	Feeling unwell a lot of time	0.57	1.45 (0.81)			1038 (93.85%)	19 (1.72%)	47 (4.25%)	2 (0.18%)	25
4	Work around the home	0.51	1.43 (0.75)			1054 (95.30%)	15 (1.36%)	36 (3.25%)	1 (0.09%)	30
5	Not being able to do the things you used to do	0.51	1.69 (0.93)			990 (89.51%)	34 (3.07%)	79 (7.14%)	3 (0.27%)	18
Cronbach’s alpha coefficient		0.74								
Psychological needs (PSY)			12.42 (13.96)	149 (13.47%)					22 (1.99%)	
6	Anxiety	0.71	1.59 (0.86)			1024 (92.59%)	20 (1.81%)	62 (5.61%)		22
7	Feeling down or depressed	0.67	1.42 (0.78)			1047 (94.67%)	15 (1.36%)	44 (3.98%)		27
8	Feelings of sadness	0.67	1.34 (0.71)			1053 (95.21%)	18 (1.63%)	34 (3.07%)	1 (0.09%)	31
9	Fears about the cancer spreading	0.67	1.71 (0.97)			981 (88.70%)	34 (3.07%)	88 (7.96%)	3 (0.27%)	17
10	Worry that the results of treatment are beyond your control	0.59	1.52 (0.88)			1010 (91.32%)	27 (2.44%)	67 (6.06%)	2 (0.18%)	20
11	Uncertainty about the future	0.67	1.68 (0.93)			992 (89.69%)	31 (2.80%)	79 (7.14%)	4 (0.36%)	18
12	Learning to feel in control of your situation	0.50	1.44 (0.88)			1003 (90.69%)	26 (2.35%)	62 (5.61%)	15 (1.36%)	23
13	Keeping a positive outlook	0.43	1.17 (0.57)			1073 (97.02%)	11 (0.99%)	21 (1.90%)	1 (0.09%)	32
14	Feelings about death and dying	0.56	1.44 (0.75)			1050 (94.94%)	16 (1.45%)	37 (3.35%)	3 (0.27%)	29
17	Concerns about the worries of those close to you	0.49	1.67 (0.75)			1039 (93.94%)	21 (1.90%)	44 (3.98%)	2 (0.18%)	28
Cronbach’s alpha coefficient		0.87								
Sexual need (SEX)			5.29 (11.46)	806 (72.88%)	2 (0.18%)				4 (0.36%)	
15	Changes in sexual feelings	0.57	1.25 (0.55)			1080 (97.65%)	10 (0.90%)	15 (1.36%)	1 (0.09%)	33
16	Changes in your sexual relationships	0.58	1.18 (0.48)			1089 (98.46%)	5 (0.45%)	10 (0.90%)	2 (0.18%)	34
31	Being given information about sexual relationships	0.30	1.21 (0.74)			1036 (93.67%)	18 (1.63%)	48 (4.34%)	4 (0.36%)	24
Cronbach’s alpha coefficient		0.64								
Patient care and support needs (PCS)			22.97 (20.46)	129 (11.66%)	5 (0.45%)				8 (0.72%)	
18	More choice about which cancer specialists you see	0.50	2.02 (1.49)			792 (71.61%)	50 (4.52%)	261 (23.60%)	3 (0.27%)	7
19	More choice about which hospital you attend	0.46	1.47 (1.06)			976 (88.25%)	19 (1.72%)	109 (9.86%)	2 (0.18%)	16
20	Reassurance by medical staff that the way you feel is normal	0.56	1.97 (1.04)			953 (86.17%)	30 (2.71%)	121 (10.94%)	2 (0.18%)	15
21	Hospital staff attending promptly to your physical needs	0.55	2.13 (1.05)			916 (82.82%)	35 (3.16%)	151 (13.65%)	4 (0.36%)	9
22	Hospital staff acknowledging, and showing sensitivity to, to your feelings and emotional needs	0.55	2.01 (1.11)			907 (82.01%)	50 (4.52%)	146 (13.20%)	3 (0.27%)	11
Cronbach’s alpha coefficient		0.76								
Health systems and information needs (HSI)			34.56 (21.46)	32 (2.89%)					20 (1.80%)	
23	Being given written information about the important aspects of your care	0.52	2.34 (1.58)			723 (65.37%)	55 (4.97%)	323 (29.20%)	5 (0.45%)	4
24	Being given information (written, diagrams, drawings) about aspects of managing your illness and side-effects at home	0.45	1.71 (1.17)			929 (84.00%)	38 (3.44%)	138 (12.48%)	1 (0.09%)	14
25	Being given explanations of those tests for which you would like explanations	0.65	2.46 (1.39)			745 (67.36%)	55 (4.97%)	303 (27.40%)	3 (0.27%)	6
26	Being adequately informed about the benefits and side-effects of treatments before you choose to have them	0.61	2.35 (1.23)			834 (75.41%)	44 (3.98%)	222 (20.07%)	6 (0.54%)	8
27	Being informed about your test results as soon as feasible	0.60	2.64 (1.36)			742 (67.09%)	43 (3.89%)	316 (28.57%)	5 (0.45%)	5
28	Being informed about cancer which is under control or diminishing (that is, remission)	0.59	2.94 (1.48)			619 (55.97%)	42 (3.80%)	437 (39.51%)	8 (0.72%)	2
29	Being informed about things you can do to help yourself to get well	0.58	2.78 (1.49)			634 (57.32%)	72 (6.51%)	396 (35.80%)	4 (0.36%)	3
30	Having access to professional counseling (e.g. psychologist, social worker, counselor, nurse specialist) if you, family or friends need it	0.46	1.68 (1.21)			913 (82.55%)	40 (3.62%)	150 (13.56%)	3 (0.27%)	10
32	Being treated like a person not just another case	0.49	2.05 (1.08)			915 (82.73%)	42 (3.80%)	145 (13.11%)	4 (0.36%)	13
33	Being treated in a hospital or clinic that is physically pleasant as possible	0.46	2.14 (1.02)			920 (83.18%)	37 (3.35%)	146 (13.20%)	3 (0.27%)	12
34	Having one member of hospital staff with whom you can talk to about all aspects of your condition, treatment and follow-up	0.56	3.15 (1.70)			476 (43.04%)	53 (4.79%)	573 (51.81%)	4 (0.36%)	1
Cronbach’s alpha coefficient		0.85								

Note: ^a^: the percentages of the floor (the lowest possible score) and ceiling (the highest possible score) of the SCNS-SF34 domain scores and the percentages of the unmet needs of each individual item were calculated. The threshold that was used for a significant floor or ceiling effect was 15%. ^b^: the ranking is based on the prevalence of moderate to high unmet need

Abbreviation: *SD* Standard deviation, *SCNS-SF34* the Short-Form Supportive Care Needs Survey Questionnaire, *PDL* Physical and daily living needs, *PSY* Psychological needs, *SEX* Sexual need, *PCS* Patient care and support needs: *HIS* Health systems and information needs: *SF-12 v2* the Short-Form-12 Health Survey version 2, *MSAS-SF* the Memorial Symptom Assessment Scale-Short Form, *HADS* the Hospital Anxiety and Depression Scale

### Factor structure and measurement invariance across gender and age groups

The original 5-factor SCNS-SF34 model was tested by confirmatory factor analysis. The goodness-of-fit indices indicted that our data fitted the original 5-factor model (RMSEA: 0.047; CFI: 0.952; TLI: 0.947). Regarding the measurement invariance across genders, the chi-square test for difference testing was 120.256 with 111 degrees of freedom, *p*-value = 0.2581. The statistical insignificance of the test supported the assumption of measurement invariance across gender groups. Regarding the measurement invariance across age groups, the chi-square test for difference testing was 113.049 with 111 degrees of freedom, *p*-value = 0.4280. The statistical insignificance of the test supported the assumption of measurement invariance across age groups. These results are shown in Table 3. The figures of the models are shown in Appendix 1.

**Table 3:** Measurement invariance of the SCNS-34

Chi-square value of model fit		DF	*p*-value	CFI^d^	TLI^d^	RMSEA^d^	90% CI
Baseline model	1762.722	517	< 0.001	0.952	0.947	0.047	0.044, 0.049
Measurement invariance across gender							
Model 1 ^a^	2240.351	1049	< 0.001	0.950	0.946	0.045	0.043, 0.048
Model 2 ^b^	2246.337	1160	< 0.001	0.954	0.956	0.041	0.039, 0.044
Chi-Square difference test for measurement invariance across gender							
	**Chi-square value**	**DF**	***p*-value**^**c**^				
	120.256	111	0.2581				
Measurement invariance across age groups							
	**Chi-square value of model fit**	**DF**	***p*-value**	**CFI**	**TLI**	**RMSEA**	**90% CI**
Model 1 ^a^	2172.758	1049	< 0.001	0.948	0.944	0.045	0.042, 0.048
Model 2 ^b^	2226.458	1160	< 0.001	0.953	0.954	0.041	0.038, 0.043
Chi-Square difference test for measurement invariance across age groups							
	**Chi-square value**	**DF**	***p*-value**^**c**^				
	113.049	111	0.4280				

Note: ^a^Model 1: it was the least constrained model. Thresholds and factor loadings were free across groups. Residual variances were fixed at one in all groups and factor means were fixed at zero in all groups. ^b^Model 2: it was the more constrained model. Thresholds and factor loadings were constrained to be equal across groups. As default, residual variances were fixed at one in the first group and freely estimated in the second group. Factor means were fixed at zero in the first group and freely estimated in the second group. ^c^: the chi-square difference value is not significant. It indicated that constraining the parameters of the nested model did not significantly worsen the fit of the model. Our result indicted measurement invariance. ^d^: Criteria for an acceptable fit were a root mean square error of approximation (RMSEA) of < 0.06, and a comparative fit index (CFI) and Tucker-Lewis index (TLI) of ≥0.90

Abbreviation: *DF* Degree of freedom, *CFI* Comparative fit index, *TLI* Tucker-Lewis Index, *SRMR* Standardised root mean square residual, *RMSEA* Root mean square error of approximation, *CI* Confidence interval

### Internal construct validity and convergent validity

The internal construct validity and convergent validity of the SCNS-SF34 were supported. The results of the analyses that evaluated internal construct validity are shown in Table 2. The item-total correlations that were corrected for overlap were > 0.4 for all items, except for item 31 (0.30). The results of the convergent validity are shown in Table 4. In line with our hypotheses, the PSY domain score had a moderate correlation with the scores of the HADS anxiety subscale (r = 0.65, *p*-value < 0.01), the HADS depression subscale (r = 0.54, *p*-value < 0.01), the MSAS psychological symptom subscale (r = 0.64, *p*-value < 0.01) and the SF-12 v2 MCS (r = − 0.46, *p*-value < 0.01). Similarly, the PDL domain score also had a moderate correlation with the MSAS physical symptom subscale (r = 0.53, *p*-value < 0.01) as well as the SF-12 v2 Physical Component Summary (r = − 0.48, *p*-value < 0.01). Other domain scores of the SCNS-SF34 only had a weak correlation with the scores of the HADS, the MASA and the SF-12 v2. The results about convergent validity are shown in Table 4.

**Table 4:** Convergent validity of the SCNS-34

	HADS Anxiety	HADS Depression	MSAS-PHY	MSAS-PSYC	SF-12 v2 PCS	SF-12 v2 MCS
Physical and daily living needs (PDL)	0.39**	0.45**	0.53**	0.44**	− 0.48**	− 0.39**
Psychological needs (PSY)	0.65**	0.54**	0.45**	0.64**	− 0.33**	− 0.46**
Sexual need (SEX)	0.14**	0.17**	0.18**	0.19**	−0.15**	−0.11**
Patient care and support needs (PCS)	0.37**	0.28**	0.23**	0.37**	−0.20**	−0.24**
Health systems and information needs (HSI)	0.35**	0.26**	0.25**	0.35**	−0.20**	−0.23**

Note: ** Pearson correlation (*p* < 0.01)

Abbreviation: *SD* SCNS-SF34: the Short-Form Supportive Care Needs Survey Questionnaire, *PDL* Physical and daily living needs, *PSY* Psychological needs, *SEX* Sexual need, *PCS* Patient care and support needs, *HIS* Health systems and information needs, *SF-12 v2 PCS* Physical Component Summary of the Short-Form-12 Health Survey version 2, *SF-12 v2 MCS* Mental Component Summary of the Short-Form-12 Health Survey version 2, *MSAS-PHYS* Physical Symptoms Subscale of the Memorial Symptom Assessment Scale-Short Form, *MSAS-PSYCH* Psychological Symptoms Subscale of the Memorial Symptom Assessment Scale-Short Form, *HADS* the Hospital Anxiety and Depression Scale

### Known-group validity

The results of the analyses that examined the sensitivity of the SCNS-SF34 are shown in Table 5**.** We first compared the SCNS-SF34 domain scores of patients who were aged ≤60 and > 60 years via an independent t-test. Statistically significant differences were found between these groups for all domains. The Cohen’s D effect size was 0.28 for the PDL domain, 0.45 for the PSY domain, 0.35 for the SEX domain, 0.41 for the PCS domain and 0.40 for the HIS domain. Second, we compared the SCNS-SF34 domain scores by gender. Statistically significant differences were found in the PSY domain (effect size: 0.33), the PCS domain (effect size: 0.38) and the HSI domain (effect size 0.32).

**Table 5:** Known-group comparison of the SCNS-34

	60 years or below		> 60 years		Cohen’s D effect Size	*P*-value
	n	Mean (SD)	n	Mean (SD)		
Physical and daily living needs (PDL)	732	16.08 (15.03)	365	12.12 (13.01)	0.28	< 0.001
Psychological needs (PSY)	723	14.36 (14.96)	359	8.49 (10.73)	0.45	< 0.001
Sexual need (SEX)	735	6.56 (12.06)	365	2.74 (9.71)	0.35	< 0.001
Patient care and support needs (PCS)	734	25.66 (20.78)	362	17.49 (18.71)	0.41	< 0.001
Health systems and information needs (HSI)	727	37.35 (20.95)	357	28.93 (21.46)	0.40	< 0.001
	Males		Females		Cohen’s D effect Size	*P*-value
	n	Mean (SD)	n	Mean (SD)		
Physical and daily living needs (PDL)	450	13.51 (13.80)	650	15.61 (14.30)	0.15	0.02
Psychological needs (PSY)	444	9.76 (11.57)	640	14.27 (15.14)	0.33	< 0.001
Sexual need (SEX)	449	5.44 (11.33)	653	5.18 (11.56)	0.02	0.69
Patient care and support needs (PCS)	447	18.50 (18.69)	651	26.04 (21.07)	0.38	< 0.001
Health systems and information needs (HSI)	443	30.53 (20.93)	643	37.35 (21.40)	0.32	< 0.001

Abbreviation: *SD* Standard deviation, *SCNS-SF34* the Short-Form Supportive Care Needs Survey Questionnaire, *PDL* Physical and daily living needs, *PSY* Psychological needs, *SEX* Sexual need, *PCS* Patient care and support needs, *HIS* Health systems and information needs

### Reliability

The value of the Cronbach’s alpha coefficient was 0.74 for the PDL subscale, 0.87 for the PSY subscale, 0.64 for the SEX subscale, 0.76 for the PCS subscale and 0.85 for the HSI subscale. Table 2 shows the results.

## Discussion

To the best of our knowledge, this was the first study to specifically evaluate and confirm the measurement invariance of the SCNS-SF34 across the main groups that might be of interest in the application of the instrument: gender and age. Measurement invariance is a prerequisite for making quantitative comparisons. In other words, confirmation of measurement invariance permits meaningful group comparisons. Our study found that the measurement model of the SCNS-SF34 as a patient-reported outcome to measure supportive care needs among cancer patients is comparable across gender and age groups.

The study also provided robust evidence in terms of validity and reliability to support the use of this instrument to evaluate the unmet supportive care needs in Chinese populations. The convergent validity of the Chinese version of the SCNS-SF34 was demonstrated in the present study. In line with our hypotheses, the PSY domain of the SCNS-SF34 had a strong-to-moderate correlation with the HADS, the MSAS-PSYCH and the scores of the SF-12 v2 MCSs. The PDL domain of the SCNS-SF34 had a moderate correlation with the MSAS-PHYS and the SF-12 v2 Physical Component Summary scores. These findings were similar to those found in Chinese patients with colorectal cancer [11], Chinese patients with breast cancer [8], Mexican patients with cancer [10], etc. Similar to previous studies, we also observed moderate correlations between the PDL domain and other psychological outcomes, such as the HADS, the MSAS-PSYCH and the SF-12 v2 MCS, which implied that unmet physical and daily living needs would lead to psychological distress. Further longitudinal studies are needed to confirm this temporal relationship. As expected, only weak correlations were found between the other domains of the SCNS-SF34 (the SEX, the PCS and the HIS) and other study outcomes because they measure different concepts.

The SCNS-SF34 was able to differentiate between age groups in all domains. We found that young patients had a higher level of unmet care needs in all five domains. Notably, other literature has also suggested that older cancer patients tend to have lower levels of unmet needs than do their younger counterparts [25, 28]. Possible explanations for the lower unmet needs among older patients include the following. A stoic attitude of older people has been found to account for age-related differences in the prevalence of physical symptoms [29]. It has been suggested that older people are inclined to keep their needs private because they believe that they should be able to manage by themselves [30]. Older people tend to underreport their needs because they do not want to burden their families and caregivers [31]. They are concerned that they will become dependent if they report problems [32]. They want to minimise their problems and needs to avoid negative stereotypes about ageing [33]. However, very few studies focus on the commonly held attitudes and beliefs towards the expression of unmet needs by older patients, warranting more studies in this area.

The SCNS-SF34 differentiated between gender groups in four out of five domains. We found that female patients reported a higher level of unmet needs. From the psycho-oncological perspective, research findings on the gender difference in cancer-related symptoms, impairment and adjustment are mixed. A validation study of German cancer patients found that female patients expressed a higher amount of unmet psychological and physical needs but a lower amount of unmet sexuality need than their male counterparts [34]. A study in Australia demonstrated that female patients reported a higher amount of unmet psychological needs than male patients [35]. Previous studies found that compared with male cancer patients, female patients reported more physical symptoms, such as nausea, vomiting and fatigue [36, 37].

Moreover, a study of more than 10,000 cancer patients found a higher rate of anxiety and depression in women than in men [38]. In contrast, some studies have found that men experience more cancer-related impairments. For example, in a study of patients with colorectal cancer in Israel, it was found that, compared with women, men reported a higher level of psychological distress, intrusive thoughts, avoidance and feelings of helplessness [39]. Another study on cancer patients in the United States found that men had significantly more cancer-related impairments, fewer social resources and more limitations in activities of daily living than did women [40]. Some theories, such as the transactional theory of stress and coping [41], can explain the gender difference in cancer burden.

A meta-analysis of 119 studies found that women experienced more stressors than did men [42]. The analysis also found that women appraised their stressors to be more severe than men did [42]. The gender difference in cancer-related distress might reflect the gender difference in distress in broader adult populations found in the meta-analysis [42]. These findings have some important implications. For example, given the gender difference in cancer-related symptoms, impairment and adjustments, it is likely that supportive care needs, preferences and expectations regarding cancer care are different between men and women [43]. Therefore, supportive care for cancer patients should be gender-specific to further improve cancer patient-centred care and to narrow the gender disparity in the cancer burden [40, 43]. In addition, research findings on women with cancer cannot be generalisable to men with cancer and vice versa.

The Chinese version of the SCNS-SF34 demonstrated good internal consistency, with a Cronbach’s alpha coefficient of > 0.7 for four domains. The Cronbach’s alpha coefficient of the SEX domain was just short of 0.7 in the current study sample, which is acceptable [44]. Notably, a previous study on Chinese patients with colorectal cancer obtained an even lower Cronbach’s alpha coefficient (0.534) [11]. One possible explanation is that item 31—‘Being given information about sexual relationships’—had a weak correlation with the other items within the same domain. Furthermore, it should be noted that the Cronbach’s alpha coefficient is also affected by the number of test items (i.e., if the number of test items is too small, it will underestimate the reliability).

The current study also serves as the foundation for further understanding of how Chinese patients with cancer prioritise supportive care. Eight of the top ten unmet needs were related to the HSI domain. Indeed, this finding is not unique to our study sample. A systematic review of studies on Chinese populations found that the HSI is the most commonly reported needs, with a pooled prevalence of 43.01% [45]. Similar to previous studies in Hong Kong [6, 8], the primary unmet need in the present study was having one member of the hospital staff with whom to talk about all aspects of one’s condition, treatment and follow-up. In Hong Kong public hospitals, patients cannot choose a doctor; they are randomly assigned to the next available doctor on the oncology team. Thus, our findings urge improvements in the continuity of cancer care in Hong Kong.

Some limitations of the present study should be noted. Given the cross-sectional nature of the analysis, we were unable to determine test–retest reliability and the responsiveness of the instrument. Also, all patients were recruited within 6 months after they completed the treatment. But that should not matter much since the findings from the current study are comparable to the one reported in previous local studies. In addition, all patients were recruited from government-funded public hospitals. Our study findings might therefore not be generalisable to private settings, where patients are more likely to have a higher socioeconomic status and better health-seeking behaviours. There might be differences in unmet needs. However, public hospitals provide cancer care for the majority of cancer patients in Hong Kong.

## Conclusion

In the present study, we evaluated the factor structure, measurement invariance, internal construct validity, convergent validity, known-group validity and internal consistency of the SCNS-SF34 among Chinese patients with cancer. Our findings suggest that the SCNS-SF34 is a valid and reliable instrument to evaluate and understand the unmet needs of Chinese cancer patients. To provide patient-centred care for cancer patients, clinicians should consider using this instrument in routine clinical practice.

## Supplementary information

**Additional file 1.** Measurement invariance across gender and age groups.

## Data Availability

The datasets generated and/or analysed during the current study are not publicly available because it contains personal data but are available from the corresponding author on reasonable request.

## Abbreviations

CFA: Confirmatory factor analysis
CFI: Comparative fit index
HADS: Hospital Anxiety and Depression Scale
HRQOL: Health-related quality of life
HSI: Health systems and information needs
MSAS-SF: Memorial Symptom Assessment Scale-Short Form
PCS: Patient care and support needs
PDL: Physical and daily living needs
PROs: Patient-reported outcomes
PSY: Psychological needs
RMSEA: Root mean square error of approximation
SCNS-SF34: Short-Form Supportive Care Needs Survey Questionnaire
SD: Standard deviation
SEX: Sexuality needs
SF-12 v2: Short-Form-12 Health Survey version 2
TLI: Tucker-Lewis index
