# Supplementary material for: Measurement invariance across gender and age groups, validity and reliability of the Chinese version of the short-form supportive care needs survey questionnaire (SCNS-SF34)
Source: Health Qual Life Outcomes. 2020 Feb 17;18:29. doi: 10.1186/s12955-020-01289-0 (PMC7027020; doi:10.1186/s12955-020-01289-0)

Measurement invariance across gender, Model 1

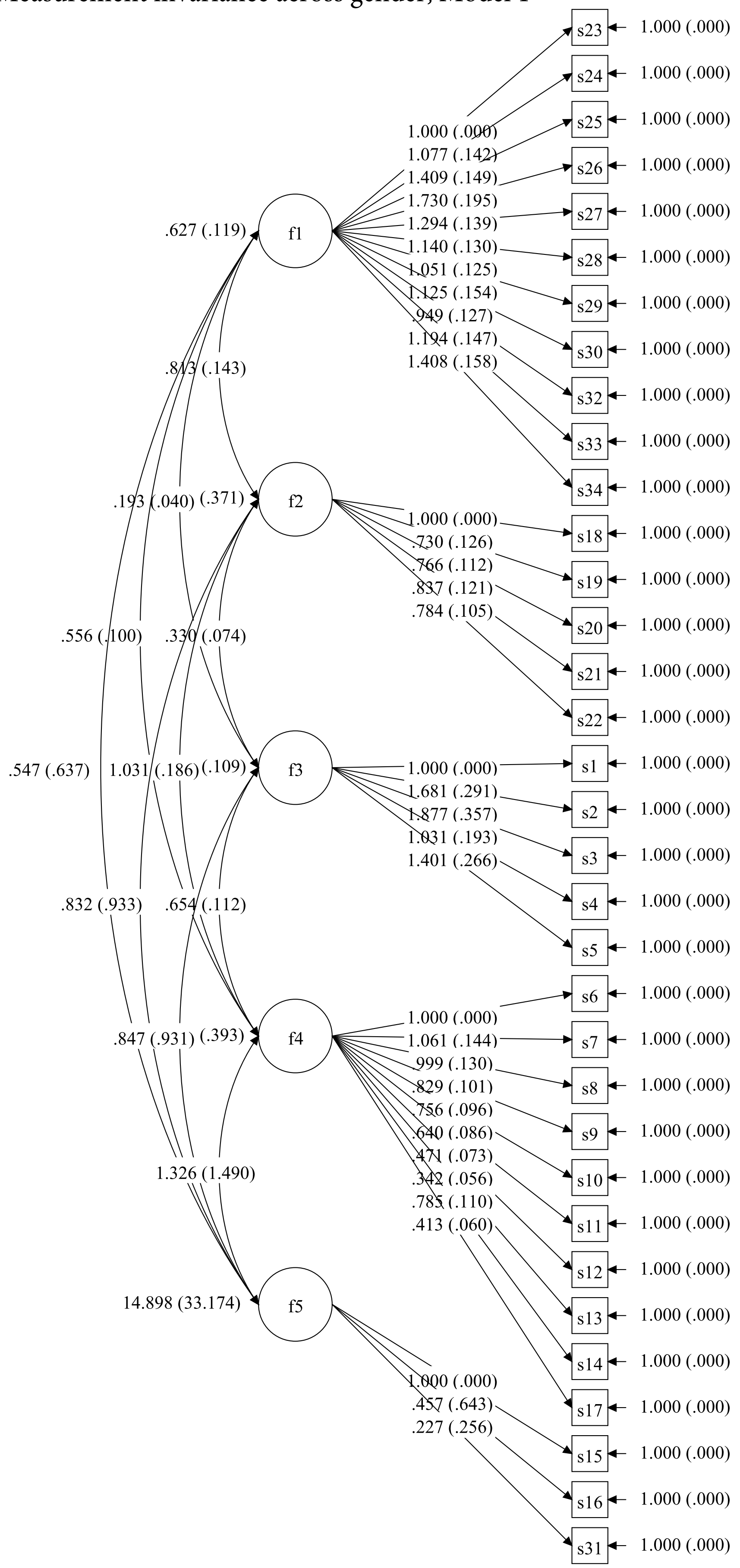

Measurement invariance across gender, Model 2

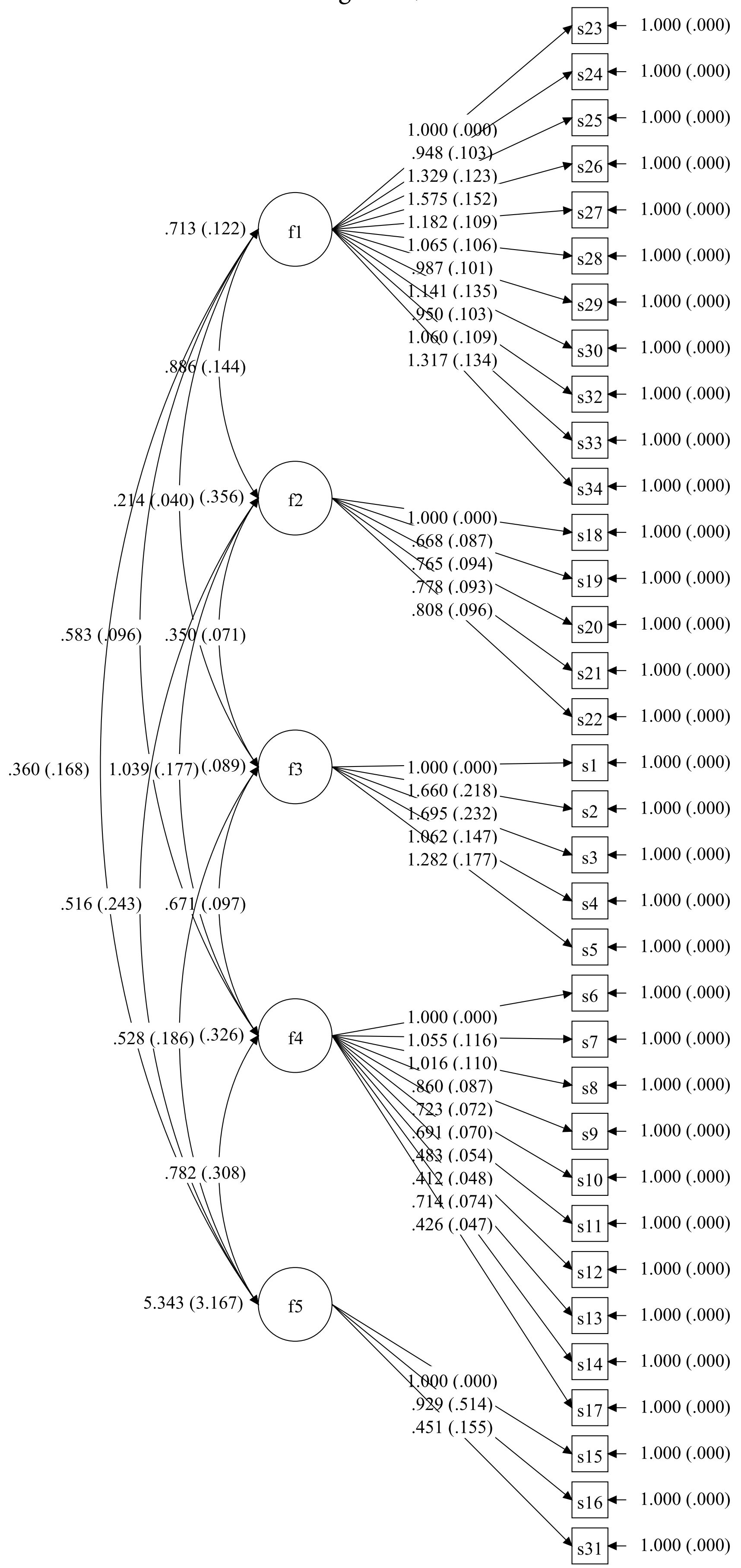

Measurement invariance across age, Model 1

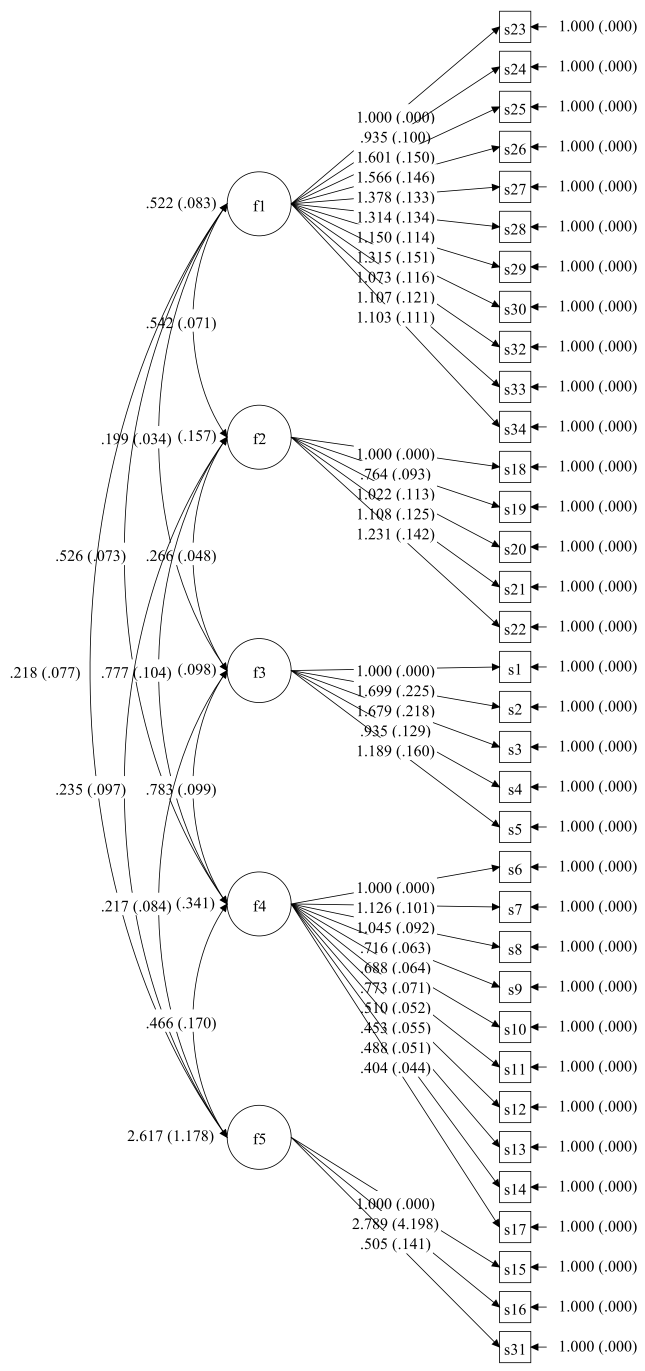

Measurement invariance across age, Model 2

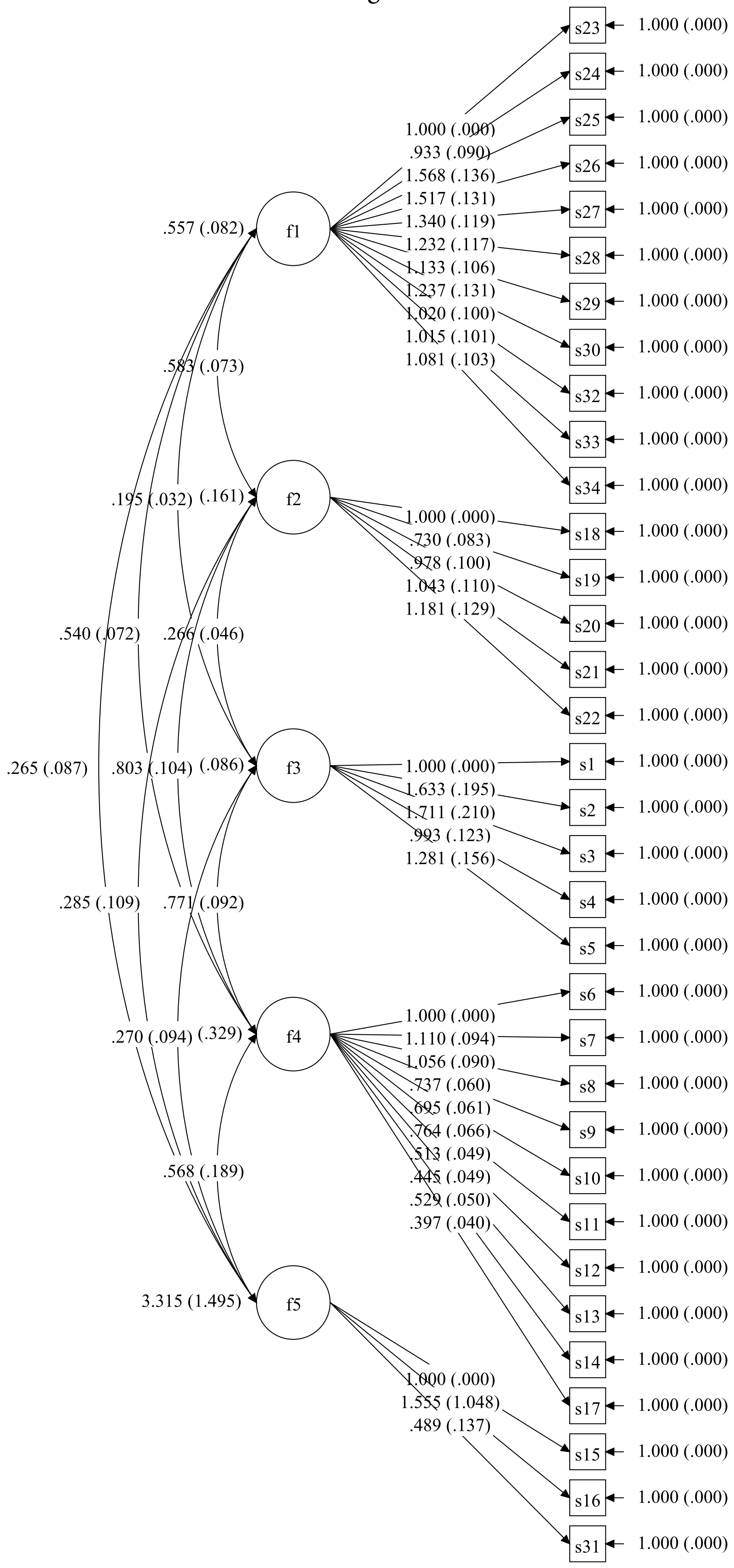

Supplement: Supplementary file 1 — Additional file 1. Measurement invariance across gender and age groups. [file 12955_2020_1289_MOESM1_ESM.pdf]
